# Postoperative delirium in patients with cancer: a narrative review of major risk factors

**DOI:** 10.1590/1516-3180.2025.0016.R2.13102025

**Published:** 2026-01-16

**Authors:** Alessandra Bittencourt de Oliveira, Adriana Mayumi Handa, Eduardo Sakai, Arthur Caus de Morais, Michael Madeira de la Cruz Quezada, Jorge Kiyoshi Mitsunaga, Assaiah Moreira Marrazzo da Costa Portugal, Eduardo Henrique Giroud Joaquim, Giane Nakamura

**Affiliations:** I Physician, Departamento de Anestesiologia, A.C.Camargo Cancer Center, São Paulo (SP), Brazil.; II Physician, Departamento de Anestesiologia, A.C.Camargo Cancer Center, São Paulo (SP), Brazil.; III Physician, Departamento de Anestesiologia, A.C.Camargo Cancer Center, São Paulo (SP), Brazil.; IV Physician, Departamento de Anestesiologia, A.C.Camargo Cancer Center, São Paulo (SP), Brazil.; V Physician, Departamento de Anestesiologia, A.C.Camargo Cancer Center, São Paulo (SP), Brazil.; VI Physician, Departamento de Anestesiologia, A.C.Camargo Cancer Center, São Paulo (SP), Brazil.; VII Physician, Departamento de Anestesiologia, A.C.Camargo Cancer Center, São Paulo (SP), Brazil.; VIII Professor, Departamento de Anestesiologia, A.C.Camargo Cancer Center, São Paulo (SP), Brazil.; IX Professor, Departamento de Anestesiologia, A.C.Camargo Cancer Center, São Paulo (SP), Brazil.

**Keywords:** Postoperative delirium, Delirium, Surgical oncology, Cancer, Aged, Malignant neoplasm, Risk factors, Older adults

## Abstract

**BACKGROUND:**

Postoperative delirium (POD) is a severe complication and the most frequent adverse event in older patients, particularly those with cancer. With the increase in the older surgical population and cancer diagnoses, the incidence of POD is expected to increase.

**OBJECTIVES:**

To identify and evaluate major risk factors for POD in patients with cancer.

**DESIGN AND SETTING:**

Narrative review conducted at the A.C.Camargo Cancer Center in São Paulo, Brazil.

**METHODS:**

PubMed, LILACS, and Embase database searches were conducted using relevant keywords from June 2023, to September 2024. We identified 279 studies; after screening and applying the eligibility criteria, 49 studies were included in the analysis.

**RESULTS AND DISCUSSION::**

POD risk factors in patients with cancer are associated with inflammation and the cumulative burden of intensive therapeutic modalities. These factors can be categorized into three domains: directly related to cancer, indirectly related to cancer, and preexisting predisposing factors. Among these factors, age is important. Additional relevant contributors include frailty, cognitive impairment, sarcopenia, pain, anxiety, and depression. A complex interaction exists between these factors that renders POD management in patients with cancer challenging; however, the impact of each factor remains unclear.

**CONCLUSIONS:**

Multiple overlapping risk factors often contribute to POD development in patients with cancer. Age is a significant risk factor, as reported in the literature. Other relevant factors have been described; however, the relative contribution of each factor to the etiology of POD remains unclear. Further research is required to address this knowledge gap.

## INTRODUCTION

 Postoperative delirium (POD) is a serious complication and the most frequent adverse event in older patients.^1,2^ POD is associated with a prolonged hospital stay, functional and cognitive impairment, increased risk of dementia, mortality, and high medical expenses.^3,4^ Furthermore, delirium is often a distressing and traumatic experience for patients and their families as well as healthcare staff.^1,5-7^

 POD is defined as a state of acute confusion characterized by fluctuating levels of attention and awareness, disorientation, disturbances in perception and memory, and disorganized thinking. Several risk factors have been identified, among which age is widely recognized as highly prominent.^5,8-11^

 Recent population aging, combined with greater access to advanced medical treatments, has led to an increase in the older surgical population.^7^ Additionally, the number of cancer diagnoses has been increasing. Globocan 2022 data revealed nearly 20 million new cancer cases, with projections estimating that annual cases will rise to 35 million by 2050, marking a 77% increase.^12^ Most patients with cancer require one or more surgeries as part of their oncological treatment. Therefore, we expect a higher number of patients with cancer and older patients with cancer in surgical centers and, consequently, a greater prevalence of POD. 

 POD has gained increasing attention in recent years and is recognized as a pertinent topic in medical research. Although the importance of POD is acknowledged, comprehensive reviews consolidating the major risk factors in patients with cancer are lacking, leading to gaps in understanding. This narrative review aimed to identify and analyze the major risk factors for POD in patients with cancer. 

## METHODS

 To conduct this narrative review, we performed a comprehensive search of the PubMed, LILACS, and Embase databases from June 2023 to September 2024 at the A.C.Camargo Cancer Center in São Paulo, Brazil. We used the following index terms (E.G. MeSH): "postoperative delirium," "delirium," "surgical oncology," "cancer," and "aged," combining them with Boolean operators "AND" and "OR." Although this study did not follow the rigorous methodology of a systematic review, a structured approach was adopted to ensure comprehensiveness and quality in the selection and analysis of studies.

**The eligibility criteria were as follows: Inclusion criteria:**
- Studies published between 2014 and 2024
- Studies involving adults (aged > 18 years)
- Studies published in English, Spanish, and Portuguese
- Systematic literature reviews, randomized clinical trials, prospective and retrospective cohort studies (with or without a control group), case reports, case series, observational research, consensus documents, and guidelines
- Full-text publications only

**Exclusion criteria:**
- Stand-alone abstracts and letters
- Opinion pieces without original data
- Unpublished studies
- Studies involving the pediatric population
- Duplicated records

The reference lists of the selected articles were screened for additional relevant publications. Study selection was conducted in a sequential process that involved the removal of duplicate records, screening of titles and abstracts, evaluation of full-text articles, and determination of final inclusion. Ethical approval was not required for this study narrative review. 

## RESULTS

**Figure 1:**
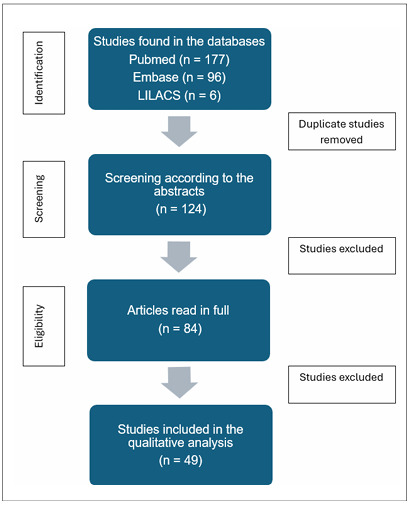
Flowchart of the study selection process.

 The initial database search yielded 279 articles. After the removal of duplicates, screening of titles and abstracts, and examination of citations, 84 studies were selected for a full-text assessment. Based on the predefined eligibility criteria, 49 studies were included in the qualitative analysis. The selection process is detailed in the flowchart shown in **Figure 1**. 

 The selected articles, along with their locations of origin, year conducted, and type, are listed in **Table 1**. Of these, 10 were systematic reviews with meta-analyses, three were meta-analyses, one was a systematic review, one was a randomized controlled trial, 13 were prospective cohort studies, 11 were retrospective cohort studies, two were consensus reports, three were guidelines, two were cross-sectional studies, two were case reports, and one was a book chapter. 

**Table 1:** Selected studies summarized by location, year, and study design

**Study**	**Location**	**Year**	**Study design**
Marcantonio^5^	USA	2017	Case report
Inouye et al.^6^	USA	2015	Case report
Aldecoa et al.^7^	International	2023	Guideline
Inouye et al.^14^	USA	2016	Prospective cohort study
Gong et al.^15^	China	2023	Systematic review and meta-analysis
Patel et al.^16^	UK	2022	Systematic review and meta-analysis
Yang et al.^17^	China	2020	Systematic review and meta-analysis
Griffin et al.^18^	UK	2020	Retrospective cohort study
Papaconstantinou et al.^19^	Greece	2023	Systematic review and meta-analysis
Hartog et al.^20^	Netherlands	2024	Prospective cohort study
Chen et al.^21^	China	2022	Retrospective cohort study
Dong et al.^22^	China	2023	Meta-analysis
Tan et al.^23^	USA	2016	Retrospective cohort study
Honda et al.^24^	Japan	2018	Retrospective study
Janssen et al.^25^	Netherlands	2019	Prospective cohort study
Wang et al.^26^	China	2019	Prospective cohort study
Wang et al.^27^	China	2024	Retrospective cohort study
Varpaei et al.^28^	USA	2024	Systematic review and meta-analysis
Sun et al.^29^	China	2023	Prospective cohort study
Mahanna-Gabrielli et al.^30^	USA	2019	Consensus report
Bush et al.^31^	International	2018	Guideline
Sadeghirad et al.^32^	Canada	2023	Meta-analysis
Lu et al.^33^	China	2023	Retrospective cohort study
Hayashi et al.^34^	Japan	2019	Retrospective cohort study
Heo et al.^35^	South Korea	2020	Prospective cohort study
Shaw et al.^36^	Canada	2022	Systematic review and meta-analysis
Tsai et al.^37^	Taiwan	2022	Prospective cohort study
Fu et al.^38^	China	2021	Meta-analysis
Handforth et al.^39^	UK	2015	Systematic review
Zhou et al.^40^	China	2024	Systematic review and meta-analysis
Tian et al.^41^	China	2023	Retrospective cohort study
Evered et al.^42^	International	2018	Consensus report
Crouch et al.^43^	USA	2023	Prospective cohort study
Harrison et al.^44^	Canada	2021	Book chapter
Regier et al.^45^	USA	2019	Prospective cohort study
Ahles et al.^46^	USA	2022	Cross-sectional study
Mandelblatt et al.^47^	USA	2018	Longitudinal cohort study
Vardy et al.^48^	Australia	2015	Prospective cohort study
Graus et al.^49^	International	2021	Consensus report
Oliveira et al.^50^	Portugal	2020	Retrospective cohort study
Mohile et al.^51^	USA	2018	Guideline
Mosk et al.^52^	Netherlands	2018	Retrospective cohort study
Makiguchi et al.^53^	Japan	2020	Retrospective study
Chen et al.^54^	USA	2024	Systematic review and meta-analysis
Falk et al.^55^	Sweden	2021	Systematic review and meta-analysis
Wada et al.^56^	Japan	2019	Prospective cohort study
Holzer et al.^57^	USA	2024	Randomized controlled trial
Kosar et al.^58^	USA	2014	Prospective cohort study
Snijders et al.^59^	Netherlands	2023	Systematic review and meta-analysis

 The final selection comprised a diverse set of study designs that reflected high-level evidence and complementary sources. This diversity provided a more comprehensive view of the research question while maintaining the methodological rigor. 

## DISCUSSION

### Definition and prevalence

 POD, as defined by the Diagnostic and Statistical Manual of Mental Disorders 5th edition, is a neurocognitive syndrome characterized by disturbed attention and reduced orientation to the environment; POD develops over a short period of time, typically within the first 3 days after surgery.^5,13^ This acute change from baseline awareness and attention often fluctuates throughout the day, and the additional cognitive disturbance is not attributable to preexisting dementia.^5,6,14^

 POD affected 5%–50% of patients, with a wide range of prevalence rates reported in the literature owing to differences in patient characteristics, surgical aggressiveness, and diagnostic methods used.^ 5,6, 15^ A substantial body of research exists in the fields of cardiac and orthopedic surgery, owing to the high complexity of these surgical procedures and the clinical conditions of the patient. In the context of cardiac surgery, delirium is recognized as the most prevalent neurocognitive complication with reported incidence rates ranging from 6%–46%.16 Similarly, in orthopedic surgery, the incidence of delirium has been reported to range from 4.5%–41.2%.^17^

 Among the surgical procedures for cancer treatment, data are typically analyzed for organ-based procedures. Esophagectomy, a complex and morbid procedure, is associated with postoperative complication rates ranging from 20%–68%.^18,19^ Papaconstantinou et al. demonstrated in a systematic review that the incidence of POD following esophagectomy ranges from 9.2%–50% and is associated with prolonged hospitalization and increased mortality rates.^19^ Other complex procedures, such as head and neck surgeries and major abdominal cancer surgeries, have been studied in recent years.^20-27^ Together, these studies have consistently shown a high incidence of POD and have highlighted its importance in patients with cancer. 

### Pathophysiology

 POD has a complex etiology and can be considered as functional cerebral decompensation resulting from multiple noxious insults that exceed the capacity of the brain for homeostasis. Decompensation is influenced by several biological factors. Although the etiology of POD cannot be reduced to a single mechanism, neuroinflammation and alterations in neurotransmitter systems are known contributing factors.^5-7^

 Neurotransmitter mechanisms involve either cholinergic deficiency or excess dopamine. This imbalance can arise from various factors, including drugs, electrolyte disturbances, metabolic derangements, hypoxia, hypercortisolism and impaired glucose oxidation. Neuroinflammation occurs simultaneously and is secondary to hypothalamic-pituitary-adrenal mediators (corticotropin-releasing hormone, adrenocorticotropic hormone, cortisol, and vasopressin) and inflammatory cytokines that cause inflammation and neuronal injury.^28,29^ These mechanisms can affect any patient; however, those with preexisting neurodegeneration, particularly older patients, and those with cognitive impairment and multimorbidity, including patients with cancer, are more severely affected. Vulnerable patients have reduced capacity to cope with adverse conditions.^5,6,30,31^

 Risk factors play crucial roles in POD development and are divided into two categories: predisposing factors, which are inherent to the patient, related to their baseline conditions, and increase vulnerability; and precipitating factors, which initiate the onset of delirium and may be reversible. The development of delirium is characterized by a complex interaction between these factors.^5-7,30^ In 2014, Inouye et al. elucidated this dynamic through a widely recognized model, demonstrating that these factors can overlap and act concurrently to influence POD.^6^

### Risk factors

 Several risk factors for POD have been identified in the literature over the past 20 years. Many studies have originated from cardiac surgery groups, and their findings are applicable to this particular population. Recent data have been published on non-cardiac surgeries and specific surgical resections for primary cancers, such as esophageal, gastric, lung, and head and neck cancers.^21,22 ,24-26 ,32-35^ Based on these findings, we compiled a summary of the most frequently cited risk factors that are directly and/or indirectly associated with POD development in patients with cancer (**Table 2**). Therefore, in this population, beyond the traditional division into predisposing and precipitating factors, risk factors can be categorized into other domains: directly related to cancer, indirectly related to cancer, and preexisting predisposing factors.^31^

**Table 2:** Risk factors: directly related to cancer, indirectly related to cancer, and preexisting predisposing factors

**Domain**	**Risk factor**
Directly related to cancer	Primary CNS tumors
	Secondary CNS tumors (brain metastases/meningeal metastases)
	Brain surgery
	Brain radiation therapy
	Chemotherapy-induced neurotoxicity
	Immunotherapy/Hormonal therapy
	Paraneoplastic neurological syndromes
	Diagnostic procedures
	Extensive resections
	Reconstructive surgeries
	Emergency surgeries
	Palliative surgeries
Indirectly related to cancer	Age
	Frailty
	Sarcopenia/Malnutrition
	Depression/Anxiety
	Pain
	Anemia
	Dehydration and electrolyte abnormalities
	Polypharmacy (including opioids and sedatives)
	Multimorbidity
	Longer hospital stay
	Sleep disturbance
	Use of restraints/Immobility
	Catheterization
	ICU admission
	Alcohol or drug abuse
	Infections
	Metabolic encephalopathy due to hepatic, renal, or pulmonary failure
Pre-exiting predisposing factors	Low educational level
	Male sex
	Visual/Hearing impairment
	History of delirium
	Preexisting cognitive impairment or dementia

CNS, Central Nervous System; ICU, Intensive Care Unit.

 These factors are discussed in the following sections, nevertheless, it is worth emphasizing that **Table 2** illustrates that patients with cancer constitute a distinct surgical population with several characteristics that may increase the likelihood of developing POD. In addition to the risk factors that can prevail before cancer diagnosis, these patients are exposed to the cumulative effects of cancer and inflammation, as well as the numerous complications and consequences of rigorous treatment modalities, including chemotherapy, radiotherapy, immunotherapy, and multiple surgeries, ranging from aggressive tumor resections to reconstructive, palliative, and emergency procedures. Beyond this context directly related to cancer, cancer predominantly affects the older population, and advanced age, a well-established and consistent risk factor, further contributes to the high incidence of POD in this population.^26,29-31^

 Aging is considered responsible for multiple brain transformations, and various theories suggest a gradual accumulation of damage to neurons, dendrites, receptors, and microglia; alterations in brain functional properties and neurotransmission; reduction in blood–brain barrier function; and anatomical disconnection between brain regions. All of these processes associated with cerebrovascular disease, along with the presence of comorbidities common with aging, may explain the vulnerability of the brain and its decreased ability to respond to stressors.^6^ Thus, advanced aging is accompanied by cognitive decline, even in the absence of neurodegenerative disease. 

 Both aging and cancer are associated with an increased prevalence of frailty. Frailty, characterized by multisystem decline, is a clinical state related to decreased reserves that results in vulnerability to stressors. Frailty develops progressively because of the accumulation of measurable clinical parameters, including comorbidities, functional impairments, and aging itself. Frailty increases the risk of adverse events such as falls, bedsores, recurrent hospital admissions, loss of autonomy, and premature death. Moreover, frailty is associated with poor postoperative outcome. Frailty is a better predictor of perioperative morbidity and mortality than is age, and is an independent risk factor for POD development.^34,36^

 Cancer and frailty are interrelated. They have a multifaceted pathophysiology, sharing common factors including metabolic and immune system dysfunction, functional and cognitive decline, multiple medication requirements, weight reduction, depression, certain comorbidities, and advanced age as a significant risk factor. More than half of older patients with cancer have prefrailty or frailty.^37^ These conditions, influenced by similar factors, increase the risk of POD development after subject to stressors such as cancer surgery and chemotherapy.^36,38 ,39^ A meta-analysis conducted on patients undergoing colorectal cancer surgeries reported nearly a 2-fold higher risk of encountering any complications, and a 3-fold higher risk of major complications, including POD.^40^ Furthermore, a retrospective cohort study conducted on patients with lung cancer revealed a significantly higher prevalence of POD in frail versus robust patients, with the risk being nearly 3-fold higher in the frail group.^41^ Consistent with these findings, Tsai et al. reported that frailty was an independent risk factor for POD in older patients with cancer undergoing elective abdominal surgery, with a 2.8 fold increase in the risk of POD occurrence.^37^

**Figure 2:**
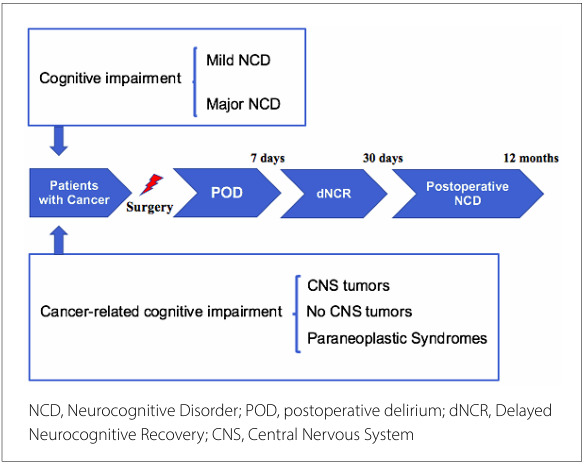
Cognitive impairment in patients with cancer and the terminology of neurocognitive disorders.

 Another key issue is precognitive impairment, a well-documented risk factor for POD in patients with cancer. Precognitive impairment is categorized into mild and major neurocognitive disorders (NCD), which are perioperative disorders. The expert panel of the Nomenclature Consensus Working Group in 2018 defined and delineated the perioperative period to establish standardized terminology for all cognitive disorders within this period.^42^ In the preoperative period, NCD are categorized into mild and major disorders. Patients with mild NCD experience cognitive decline with minimal functional impairment, whereas those with major NCD experience significant cognitive impairment that significantly affects their daily activities. Both patients were underdiagnosed in the preoperative period. In the postoperative period, delineation includes POD occurring within hours to one week post-procedure or until discharge, whichever occurs first, and long-lasting cognitive decline diagnosed up to 30 days (delayed neurocognitive recovery) and up to 12 months after the procedure (postoperative NCD) (**Figure 2**). 

 Beyond this established terminology, precognitive impairment in patients with cancer can be divided into those with and without central nervous system (CNS) tumors, collectively referred to as cancer-related cognitive impairment (**Figure 2**). Previously known as "chemobrain," cancer-related cognitive impairment encompasses subjective and objective changes in cognitive function that can occur before, during, and after cancer treatment.^43^

 In patients without CNS tumors, precognitive impairment appears multifactorial, and the underlying mechanisms remain unclear. The major hypotheses are related to the neuroimmune and neuroinflammatory changes caused by tumors and cancer treatment, primarily chemotherapy. In patients with CNS tumors, which could be either primary brain tumors or secondary metastases, precognitive impairment is directly related to brain tumors and their treatment, including radiation and/or brain surgery.^43,44^

 Cancer-related cognitive impairment affects up to 75% of patients with cancer and can persist long after treatment completion.^43^ The main symptoms reported include visual and memory impairment, learning and attention deficits, executive functioning decline, and difficulty in processing new information and multitasking. These symptoms can negatively impact quality of life, level of independence, decision-making abilities, treatment compliance, and consequently work resumption and daily activities.^44,45^

 Most studies on cancer-related cognitive impairment have primarily focused on patients with breast cancer. Ahles et al.^46^ demonstrated that breast cancer survivors exhibited lower cognitive performance and higher levels of frailty than did controls. Similarly, in a multisite prospective "thinking and living with cancer study" Mandelblatt et al.^47^ demonstrated that older cancer survivors experienced decreased cognitive function scores. These findings have been confirmed in patients with colorectal cancer. Vardy et al.^48^ reported that the rates of cognitive impairment in patients with localized colorectal cancer ranged from 36%–52% between baseline and 24 months, compared to 6%–19% in healthy patients without cancer. 

 Considering the role of paraneoplastic neurological syndromes in patients with cancer is crucial, as they may contribute to cancer-related cognitive impairment. Paraneoplastic neurological syndromes are a group of disorders that are not directly caused by brain tumors or the side effects of cancer treatment. These are immune-mediated disorders of the peripheral or central nervous system that are frequently associated with autoantibodies against neural antigens expressed by tumors, resulting in severe neurological deficits. Paraneoplastic neurological syndrome has varying clinical presentations associated with a characteristic spectrum of antibodies and often manifests as severe and well-defined neurological symptoms, the most common of which are subacute cerebellar degeneration, sensory neuropathy, and limbic encephalitis. Although the prevalence of this condition is reported to be rare in some studies, with percentages ranging from less than 0.01%1% in patients with cancer, it is becoming increasingly common owing to advances in medical treatment and significantly improved survival rates.^49 ,50^

 These findings emphasize the importance of evaluating cognitive function and the potential risks of developing cancer-related cognitive impairment and POD before initiating therapy, as recommended by the American Society of Clinical Oncology guidelines.^51^

 Cancer, age, frailty, and cognitive performance are dynamically interrelated; they share many biological pathways that are usually present concomitantly and act synergistically, making perioperative patient management challenging. 

 Other significant risk factors for POD have been identified in the literature, including sarcopenia, depression, anxiety, and pain. 

 Sarcopenia, characterized by a decrease in skeletal muscle mass, muscle power, or physical activity, is prevalent in patients with cancer and is considered a significant risk factor for POD in patients with colorectal cancer undergoing surgery. The association between sarcopenia and POD is strong in patients with malnourishment and physical dependency.^52^ The interplay between several factors, such as frailty, malnourishment, inadequate food intake, advanced age, and changes caused by disease, surgery, and treatment, leads to a vicious cycle of muscle loss and weakness. Additionally, sarcopenia was a significant independent risk factor for hypoactive and mixed-type POD in oral cancer surgery.^53^ These findings emphasize the relevance of sarcopenia, nutrition, and rehabilitation and highlight the need for further studies involving various oncological surgery types and patients. 

 Depressive and anxiety symptoms are more prevalent in patients with cancer than in the general population, with one in four patients with cancer experiencing depression.^54^ In addition to affecting quality of life and treatment adherence, depression and anxiety symptoms are associated with POD and poor surgical outcomes.^55,56^ Although the exact underlying mechanisms remain unclear, inflammatory cytokines may be involved. A systematic review and meta-analysis conducted in 2020 revealed the impact of depression on POD following cardiac surgery. Moreover, a prospective observational cohort study demonstrated that anxiety in patients with cancer was a predictive factor for POD.^ 55,57^ Recognizing the implication of the relationship between mental health and cancer is crucial because improving perioperative mental health may have a substantial impact on surgical outcomes in such patients. 

 Preoperative pain is independently associated with the development of POD.^58^ Pain is a common experience among patients with cancer, and most older adults require pain management at some point during their care.^59^ Furthermore, pain is strongly associated with depressive symptoms. Similar to previous findings, a study on general elective surgeries indicated that patients with depressive symptoms are more likely to report severe pain and develop delirium.^58^ Patients with cancer experience a significant increase in pain and depressive symptoms, making this a serious clinical issue. 

 In addition to the risk factors described, several others have been listed in the literature, including anemia; dehydration; electrolyte abnormalities; polypharmacy (including opioids and sedatives); multimorbidity; longer hospital stay; ICU admission; sleep disturbance; use of restrains or immobility; catheterization; alcohol and drug abuse; infections; metabolic encephalopathy due to hepatic, renal, or pulmonary failure; low educational level; male sex; visual or hearing impairment; and history of delirium.^5-7,30,32^

 Therefore, perioperative management of patients with cancer is challenging because of the complex interactions between multiple factors that are often present and act synergistically. Delirium treatment is complex. Non-pharmacological interventions, including reorientation, early nutrition and mobilization, and the use of personal sensory aids such as glasses and hearing aids, are the primary approaches for managing delirium. Additionally, early catheter removal and identification of potential triggers, such as pain, hypoxia, infection, or bladder distention, with the assistance of a multidisciplinary team, are crucial. Given the poor response observed, pharmacological treatment is typically reserved for agitated patients. It is estimated that 30%–40% of delirium cases are preventable, highlighting the importance of proactive prevention measures.^ 7,15,28^ Moreover, patients often require adjuvant treatment after surgery, which should not be delayed. Therefore, it is essential for patients to resume their activities and treatment as early as possible. 

## CONCLUSION

 POD is a severe complication that is particularly prevalent among patients with cancer and can result in significant morbidity. In light of the prevalence of POD along with morbimortality, high costs, and difficulties involved in the treatment of this complication, preventing up to 30%–40% of its incidence should be a crucial consideration. Furthermore, most patients with cancer require adjuvant treatment following surgery; therefore, it is imperative that these patients return to their normal activities and treatment with minimal delay. Consequently, prioritizing actions to enhance modifiable factors, improve preoperative conditions, and cultivate high cognitive reserve and physical status can ultimately increase resilience against potential stressors, thereby reducing the incidence of POD. 

 Although the literature on POD is expanding, a major gap remains in understanding the specific impact of individual risk factors on the development of POD. Patients with cancer frequently present with multiple concurrent risk factors, such as advanced age, frailty, and preexisting cognitive impairment. However, the relative contribution of each factor to the etiology of POD remains unclear, and its delineation is complex and requires further clarification. Therefore, additional research is required to address this knowledge gap. 

## Data Availability

Data supporting the findings of this study are available from the corresponding author, Alessandra Bittencourt de Oliveira, upon reasonable request.
